# From the Cosmos to the Cell: The Central Role of Iron in the Chemistry and Evolution of Life

**DOI:** 10.3390/ijms27156651

**Published:** 2026-07-25

**Authors:** Paolo Arosio, Fadi Bou-Abdallah

**Affiliations:** 1 Department of Molecular and Translational Medicine, University of Brescia, 25121 Brescia, Italy; 2 Department of Chemistry, State University of New York (SUNY) at Potsdam, Potsdam, NY 13676, USA

**Keywords:** iron homeostasis, iron cofactors, iron–sulfur clusters, heme biosynthesis, redox chemistry, great oxidation event, origin of life, iron metabolism, prebiotic chemistry, biological evolution

## Abstract

Iron, with the unique stability of its nucleus, occupies an unusual position among the elements: its abundance on Earth is not simply a geological accident but a direct consequence of nuclear reactions that happened inside stars billions of years ago. Formed at the final stages of fusion in stars, iron spread through space by supernova explosions and became part of the material that formed Earth, eventually becoming the dominant component of the planet’s core. At the surface, iron’s redox chemistry shaped the early atmosphere and oceans, and its availability as a soluble ferrous ion in the anaerobic Archean ocean made it a natural cofactor for the first enzymatic reactions. That same redox flexibility and the ability of iron to shuttle between Fe^2+^ and Fe^3+^ across a wide range of electrochemical potentials explain why virtually every major metabolic pathway in biology depends on iron in one form or another. Yet iron is also dangerous: free and chelated iron can catalyze the production of toxic hydroxyl radicals through Fenton chemistry, the reactivity of which depends strongly on the nature of the chelating ligand, and every living system must balance its need for iron against the oxidative damage that uncontrolled iron causes. This tension between catalytic necessity and chemical toxicity has driven much of the regulatory complexity we observe in modern iron metabolism. In this review, we first outline iron’s journey from its formation in stars to its role in shaping Earth’s structure and the emergence of early iron-dependent biology. We then discuss in detail how fundamental physical and chemical factors continue to influence living systems.

## 1. Introduction

Iron is, in many respects, the element that connects the physics of stars to the chemistry of cells. Forged in the cores of massive stars and then dispersed into interstellar space by supernova explosions hundreds of millions of years ago, iron then accreted into the early Earth, differentiated into the planet’s core, cycled through the oceans and atmosphere, and was ultimately recruited by the first living systems as a redox catalyst [1]. Following iron across these scales offers a useful way of thinking about how fundamental physical constraints, in this case, the nuclear stability of iron-56 and the electrochemical properties of the Fe^2+^/Fe^3+^ couple, propagate upward from astrophysics into geochemistry and biology.

**Figure 1:**
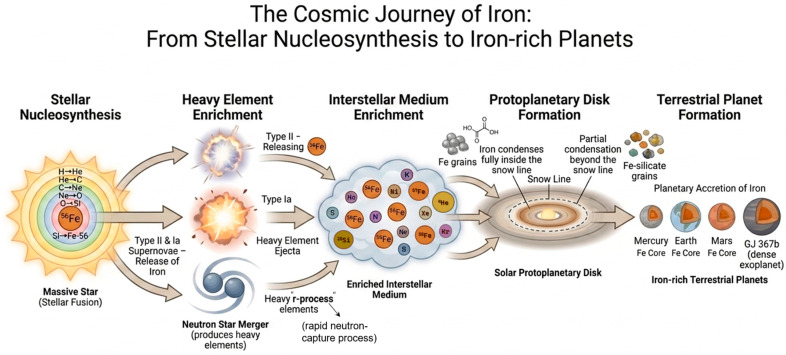
The cosmic journey of iron. Iron-56 forms as the final product of stellar fusion in massive stars and is released into space through Type II and Type Ia supernovae, with additional heavy elements contributed by neutron star mergers. This dispersed iron enriches the interstellar medium and later condenses in the solar protoplanetary disk, fully inside the snow line and partially beyond it, leading to the formation of iron-rich terrestrial planets such as Mercury, Earth, Mars, and the dense exoplanet GJ 367b. This figure was generated with the assistance of the AI-based tool FigureLabs.

Iron-56 has the highest binding energy per nucleon of any nucleus, approximately 8.8 MeV, which means that stellar fusion reactions become energetically unfavorable once they reach iron [2]. A massive star that has built up an iron core cannot continue releasing energy by fusing it into heavier elements; instead, the core collapses under gravity, and the resulting supernova disperses iron throughout the surrounding interstellar medium [3]. This process has been repeating for most of the history of the universe, progressively enriching galaxies with iron, and it explains why iron is among the most abundant heavy elements in the cosmos (Figure 1). On Earth, iron accounts for roughly 32% of the planet’s mass, more than any other element, and its concentration in the planet’s interior has had consequences for surface habitability that may not be fully appreciated.

At the Earth’s surface, iron’s geochemical behavior is dominated by its redox chemistry. In the absence of oxygen, ferrous iron (Fe^2+^) is soluble and was present in the Archean ocean at millimolar concentrations, providing early microorganisms with ready access to a versatile transition metal. The advent of oxygenic photosynthesis changed this fundamentally: as cyanobacteria began producing O_2_, roughly 2.4 billion years ago, dissolved Fe^2+^ was oxidized to insoluble Fe^3+^ and precipitated from the oceans as the banded iron formations that now constitute some of the world’s largest iron ore deposits. This Great Oxidation Event [4] effectively depleted the ocean of bioavailable iron and imposed new selective pressures on life: organisms had to evolve high-affinity iron acquisition systems, iron storage proteins, and regulatory mechanisms to manage iron homeostasis in an increasingly iron-limited world.

The biological consequences of this transition are still visible in the biochemistry of modern cells. Bacteria produce siderophores, which are small and high-affinity iron chelators, to scavenge iron from insoluble minerals or compete with other organisms for dissolved iron. Eukaryotes have sophisticated endocytic pathways for taking up iron bound to transferrin, and they store excess iron in the hollow nanocage of ferritin, a protein that can accommodate up to 4500 iron atoms in a biomineral core. In vertebrates, systemic iron homeostasis is coordinated by the peptide hormone hepcidin, which controls iron absorption and recycling by regulating the expression of ferroportin, the only known iron export protein. These systems collectively reflect the extraordinary evolutionary pressure that iron limitation has placed on living organisms over the past 2.4 billion years.

Alongside these regulatory challenges, iron’s chemistry also makes it one of the most powerful catalysts in biology. The Fe^2+^/Fe^3+^ redox couple spans a potential range of approximately −0.5 to +0.4 V depending on the coordination environment, encompassing the mid-range of biological electron transfer reactions [5]. Iron–sulfur clusters participate in a remarkably wide range of biological functions far beyond electron transfer, including energy conversion in both respiration and photosynthesis, DNA replication and repair, RNA modification, amino acid and cofactor biosynthesis, gene regulation, and signal transduction, making them arguably the most functionally versatile of all biological cofactors; heme iron binds and activates O_2_ in cytochromes, peroxidases, and oxygen-carrying proteins; non-heme mononuclear iron centers activate O_2_ for challenging oxidative reactions in dioxygenases. In nitrogenase, a complex iron-molybdenum cofactor reduces N_2_ to ammonia at ambient temperature and pressure, a reaction that abiotic chemistry cannot replicate under physiological conditions. The range of chemistry that iron enables in biology is arguably wider than that of any other element.

This review is organized around iron’s progression through increasing levels of biological complexity, beginning with its astrophysical origins and ending with its role in the evolution of life. We have tried to write it as an account of the physical and chemical logic that links these domains, rather than as a catalog of iron-containing proteins or geological iron phases. Our aim is to show that iron’s recurrent importance across such disparate systems reflects genuine underlying constraints that, once understood, make many features of iron biochemistry and geochemistry seem less arbitrary than they might otherwise appear.

## 2. Cosmic Origins of Iron

### 2.1. Nuclear Stability and the Stellar Origin of Iron

Iron’s cosmic abundance has a simple physical explanation: iron-56 is the most tightly bound nucleus that exists. With a binding energy of approximately 8.8 MeV per nucleon, slightly higher than nickel-62, which is technically the most stable by some measures, iron represents the terminus of exothermic nuclear fusion [2,3]. Stars generate energy by fusing lighter elements into heavier ones, and they can continue doing so only as long as each fusion reaction releases net energy. When a massive star has converted its core to iron, further fusion reactions would consume rather than release energy, and the star loses its pressure support against gravitational collapse [6]. The result is a core collapse supernova, which disperses the star’s iron-rich material into the interstellar medium [7].

Iron is synthesized during the late stages of stellar evolution, following successive burning phases (hydrogen through silicon), which produce progressively heavier elements. By the final stages, the star develops a layered structure with an iron-rich core [3,8]. When this core becomes unstable, collapse occurs rapidly, triggering a supernova.

Both core-collapse supernovae and thermonuclear (Type Ia) supernovae contribute to the cosmic iron inventory, with Type Ia events being the dominant source over long timescales. As generations of stars form and die, iron accumulates in the interstellar medium, leading to a steady increase in metallicity ([Fe/H]) over cosmic time [9,10]. This progressive enrichment governs the availability of iron in planetary systems and ultimately in the materials that support biological processes (Figure 1).

### 2.2. Iron in Planetary Formation

The solar system formed from a molecular cloud with solar metallicity, so iron was abundant from the outset of planet formation [11]. As the solar nebula cooled, iron condensed at relatively high temperatures, forming metallic iron and iron sulfides among the first solid phases. These iron-rich grains were incorporated into planetesimals and ultimately into the terrestrial planets. The distribution of iron across planets reflects both their formation distance from the Sun and the extent of metal–silicate separation during accretion and differentiation.

The terrestrial planets show marked differences in iron content. Mercury is exceptionally iron-rich (~70% by mass), possibly due to mantle loss during a giant impact or formation from iron-enriched material [12]. Earth and Venus contain ~32% iron, largely concentrated in their cores. Mars (~25% iron) formed under more oxidizing conditions and followed a distinct thermal and differentiation history. The Moon, at ~8% iron, is strongly depleted, consistent with formation from silicate-rich material ejected during a giant impact [13,14].

Meteorites provide direct evidence of early solar system processes. Iron meteorites represent the metallic cores of differentiated bodies, while chondrites preserve primitive compositions and record redox conditions in the protoplanetary disk. Their diversity reflects the wide range of environments in which planetary materials formed. Exoplanet studies extend this framework whereby stars with higher iron abundances are more likely to host planets, particularly rocky ones [15,16]. Some exoplanets exhibit densities exceeding Earth’s, consistent with iron-rich compositions, indicating that planetary iron content can vary widely across planetary systems [17].

In summary, the astrophysical history of iron set the stage for its dominance in planetary chemistry, and ultimately in biochemistry. The same nuclear property that makes iron the terminus of stellar nucleosynthesis, its exceptional stability, also makes it thermodynamically resistant to further transformation under geological conditions, ensuring its long-term availability as a geochemical and biological resource. Next, we will briefly discuss how iron shaped the early Earth.

## 3. Iron and the Early Earth

### 3.1. Core Formation and the Geodynamo

Shortly after Earth formed (~4.5 billion years ago), metallic iron segregated from the mantle and sank to form the core in a process often termed the Great Iron Catastrophe [18]. This differentiation, occurring within the first 30–100 million years, released substantial gravitational energy, driving large-scale melting and the formation of a magma ocean [19]. The remaining mantle retained a smaller fraction of iron (~8% FeO), while most iron became concentrated in the core.

Today, the core consists of a solid inner core and a convecting liquid outer core composed primarily of iron and nickel with lighter elements [20]. Convection in the electrically conducting outer core sustains the geodynamo, which generates Earth’s magnetic field [21]. This magnetic field plays a central role in long-term habitability by shielding the atmosphere from the solar wind and cosmic radiation. In contrast, Mars lost its magnetic field early, likely due to rapid core cooling, leading to atmospheric loss and surface conditions hostile to life. This contrast highlights how the distribution and state of planetary iron can determine whether a planet remains habitable.

### 3.2. Iron in the Crust and Mantle

Above the core, iron is distributed through the mantle and crust in silicate and oxide minerals. In the mantle, it occurs mainly as Fe^2+^ in olivine, pyroxene, and garnet (~8% FeO), where it influences melting behavior, viscosity, and electrical conductivity, thereby affecting mantle convection and Earth’s thermal evolution [22].

**Figure 2:**
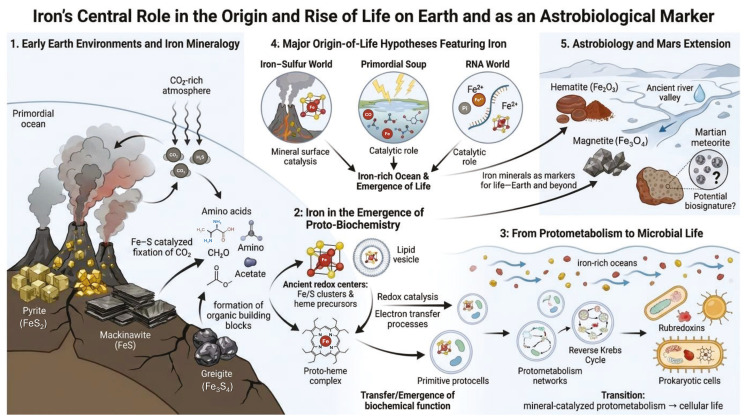
Iron’s central role in the origin and rise of life. Illustration showing how iron-rich early Earth environments, especially hydrothermal vents, catalyzed prebiotic chemistry through Fe–S minerals, promoting the formation of Fe/S clusters, heme-like complexes, and early protocells. The figure traces the transition from mineral-based protometabolism to the first microorganisms in iron-rich oceans and highlights major origin-of-life hypotheses (Iron–Sulfur World, Primordial Soup, and RNA World). Mars is included to show iron oxides as potential indicators of past water and biosignatures, emphasizing iron’s catalytic and astrobiological significance. This figure was generated with the assistance of the AI-based tool FigureLabs.

In the crust, iron is the fourth most abundant element, averaging ~3.5% in continental crust and ~8% in oceanic crust. Its speciation depends on redox conditions: Fe^2+^ in ferrous silicates and carbonates (siderite and FeCO_3_), Fe^3+^ in ferric oxides and hydroxides (hematite, Fe_2_O_3_; goethite, FeOOH), and as mixed-valence Fe^2+^/Fe^3+^ in magnetite (Fe_3_O_4_) [23]. Weathering of iron-bearing rocks releases iron into soils and rivers, ultimately supplying the oceans, where it plays a key role in regulating marine primary productivity (Figure 2).

### 3.3. Banded Iron Formations and the Great Oxidation Event

Banded iron formations (BIF) are a major geological record of iron cycling on the early Earth, consisting of alternating iron-rich (i.e., hematite and magnetite) and silica-rich layers. Deposited mainly between ~2.8 and 1.8 billion years ago, they reflect the interaction between abundant dissolved Fe^2+^ in Archean oceans and oxidizing processes that converted it to insoluble Fe^3+^ [24]. Likely oxidants include microbial photoferrotrophy and possibly abiotic photo-oxidation, indicating a close link between iron chemistry and early metabolic activity.

The Great Oxidation Event marked a major shift in Earth’s redox state as oxygenic photosynthesis increased atmospheric O_2_. This led to rapid oxidation and removal of Fe^2+^ from surface waters, driving peak BIF deposition and its subsequent decline as oceanic iron became depleted [25]. This transition imposed strong evolutionary pressure on iron metabolism. As soluble iron became scarce, organisms evolved high-affinity uptake systems such as siderophores, iron storage proteins like ferritin, and regulatory networks (e.g., Fur and IRE-IRP) to maintain iron homeostasis. These adaptations remain central to modern biology.

## 4. Iron in Biology

### 4.1. Iron at the Origin of Life

It is difficult to imagine early metabolism without iron. In the anoxic Archean ocean, ferrous iron was abundant, soluble, and chemically reactive, providing catalytic potential before protein enzymes evolved. The iron–sulfur world hypothesis proposed by Günter Wächtershäuser suggests that proto-metabolic reactions emerged on iron–sulfur mineral surfaces at hydrothermal vents, where redox reactions such as FeS oxidation could have driven CO_2_ reduction to organic molecules [26]. Even if the exact chemistry remains debated, iron–sulfur surfaces are widely regarded as plausible bridges between geochemistry and early metabolism (Figure 2).

Hydrothermal vent systems offer conditions favorable for prebiotic chemistry, including strong redox and pH gradients, mineral catalysts, and continuous fluxes of H_2_, CO_2_, and H_2_S. In modern alkaline vents such as the Lost City Hydrothermal Field in the Atlantic, H_2_-rich and high-pH fluids could have supported CO_2_ reduction via the Wood–Ljungdahl pathway, a deeply conserved carbon fixation route that depends heavily on iron–sulfur cofactors. Iron- and nickel-sulfide minerals in vent walls likely served as inorganic precursors to modern metalloenzymes [27].

The widespread presence of iron–sulfur clusters in ancient and conserved enzymes further supports their early origin. They occur in ferredoxins, which are among the smallest and most structurally simple electron carriers known; in the active sites of nitrogenase, pyruvate ferredoxin oxidoreductase, and other enzymes involved in core metabolic pathways; and in radical SAM enzymes, which catalyze a remarkably diverse array of reactions through a mechanism involving a [4Fe-4S] cluster. If we take the distribution of Fe-S clusters across the tree of life as evidence of their antiquity, it is hard to avoid the conclusion that iron–sulfur chemistry was central to life from its earliest stages, before the evolution of heme, before the divergence of the major domains of life, possibly even before the evolution of protein-based catalysis [28] (Figure 2).

Iron–porphyrin compounds have also been identified in carbonaceous meteorites, suggesting that heme-like chemistry may have been available in the prebiotic environment before life originated [29]. Whether such compounds played any role in earlier life or were simply incorporated into proto-biological systems as they became available is unknown. In modern biology, this chemistry expanded into diverse functions, including respiration (cytochromes), oxygen transport (hemoglobin), oxidative stress defense (catalase), and detoxification (cytochrome P450). This diversity reflects how a single iron-based scaffold became central to biological innovation (Figure 2).

### 4.2. Why Does Biology Use Iron?

Among the transition metals, iron has several properties that make it particularly well suited for biological catalysis. Its redox potential in aqueous solution (~−0.5 to +0.4 V) depends on its ligand environment and oxidation state, which places it squarely in the middle of the range of reduction potentials relevant to biological electron transfer [5]. This is important because metabolic electron transfer chains function by passing electrons through a series of carriers with progressively more positive reduction potentials, driving the reduction of O_2_ (E_0_′ = +0.82 V) or other terminal acceptors. Iron-containing carriers (e.g., ferredoxins, iron–sulfur clusters in complex I and II, and cytochromes) are found throughout these chains in organisms from bacteria to mammals, reflecting iron’s suitability across the full range of biologically relevant potentials.

Iron’s coordination chemistry is also unusually flexible. It can adopt octahedral, tetrahedral, square planar, and other geometries; it can bind ligands through nitrogen, oxygen, or sulfur donors; and it can exist in multiple spin states (high spin and low spin for both Fe^2+^ and Fe^3+^ that have distinct reactivity profiles [30]. This flexibility allows evolution to fine-tune the redox potential and reactivity of iron-containing active sites over a wide range by changing the identity and geometry of the coordinating ligands. The roughly 2000 iron-containing proteins identified in current databases span functions as diverse as electron transfer, oxygen transport, oxygen activation, radical generation, hydrolysis, and sensing, encompassing a breadth of biochemical roles that no other metal approaches.

The other side of iron’s utility is its tendency to generate reactive oxygen species. In the presence of H_2_O_2_, Fe^2+^ undergoes Fenton chemistry to produce the hydroxyl radical (OH^.^), which is among the most reactive species in aqueous chemistry and attacks DNA, proteins, and lipids with near-diffusion-limited rate constants [31]. This chemistry is not merely a laboratory curiosity: it is the basis of oxidative DNA damage in living cells, and it is why essentially all organisms maintain mechanisms to minimize the concentration of reactive iron species. It is important here to distinguish between (i) thermodynamically free aquated Fe^2+^, which is estimated to be below 10^−18^ M in yeast, a value reflecting the extraordinarily tight thermodynamic control over unliganded iron; (ii) the chelatable or labile iron pool (LIP), a kinetically accessible fraction of low-molecular-weight iron complexes typically in the low micromolar range that is detectable by fluorescent chelator probes and represents the principal driver of Fenton reactivity in vivo; and (iii) protein-bound iron, which constitutes the vast majority of cellular iron and is functionally inert with respect to Fenton chemistry under normal conditions. While the labile iron pool in transit is tightly regulated, the protein-bound iron is not free in cells either; it moves from one protein-bound form to another, chaperoned by iron-binding proteins. The toxicity of iron overload conditions such as hereditary hemochromatosis reflects what happens when these regulatory systems fail, and iron accumulates to levels that overwhelm the cell’s antioxidant defenses.

The following subsections survey the three major structural solutions biology has found for deploying iron’s reactivity (Section 4.2.1), then examine how these solutions are applied in the most iron-intensive metabolic systems, photosynthesis (Section 4.2.2), aerobic respiration (Section 4.2.3), the specialized chemistry of individual cofactor classes (Section 4.2.4, Section 4.2.5 and Section 4.2.6), as well as reactive oxygen species management and antioxidant defense systems (Section 4.2.7).

#### 4.2.1. Iron Cofactors: Three Solutions to the Same Problem

Iron enters biological catalysis through three distinct types of cofactor: heme groups, iron–sulfur clusters, and non-heme iron centers, each representing a different structural solution to the problem of deploying iron’s reactivity in a controlled way. The diversity of these architectures, and the fact that all three are ancient and phylogenetically widespread, suggests that iron cofactor chemistry had multiple independent origins in the prebiotic or early biotic world, and that different lineages then elaborated these starting points in different directions.

Heme is a porphyrin macrocycle with iron at its center, coordinated through four nitrogen atoms from pyrrole rings. The extended pi-conjugation of the porphyrin ring system creates a hydrophobic, electronically tunable environment around the iron, and the protein scaffold that surrounds the heme provides additional steric and electrostatic control [32]. Subtle variations in heme structure such as differences in the peripheral substituents on the porphyrin ring, the identity of the axial ligands provided by the protein, and the polarity of the binding pocket give rise to heme proteins with vastly different properties: from the tight, reversible O_2_ binding of hemoglobin and myoglobin, to the powerful oxidizing chemistry of cytochrome P450, to the rapid electron transfer of cytochrome c [32,33]. The same cofactor, in different protein environments, performs fundamentally different chemistry.

Iron–sulfur (Fe-S) clusters are inorganic complexes in which iron atoms are bridged by sulfide ions and typically anchored to the protein by cysteine thiolate ligands [34,35]. The simplest [2Fe-2S] clusters consist of two iron atoms and two bridging sulfides; more complex [3Fe-4S] and [4Fe-4S] clusters add additional iron and sulfide atoms to create structures with a greater range of redox states and catalytic capabilities. Fe-S clusters are among the most phylogenetically ancient cofactors known and appear in some of the most conserved enzymes across all three domains of life. Fe-S clusters can be assembled spontaneously from iron and sulfide under reducing conditions in vitro, and this abiotic reactivity is consistent with an origin in the iron- and sulfur-rich environment of early hydrothermal systems; however, in modern living cells, Fe-S cluster assembly is strictly enzymatic, mediated by dedicated machineries, the ISC (iron–sulfur cluster) system under normal conditions [34] and the SUF system under iron-limiting or oxidative stress conditions [35]. Their chemical instability in the presence of oxygen, which can destroy the cluster by oxidizing its sulfide components, represents both a vulnerability and, in some cases, a feature. For instance, certain Fe-S proteins use cluster degradation as a sensing mechanism to detect oxidative stress or iron depletion and adjust gene expression accordingly.

**Figure 3:**
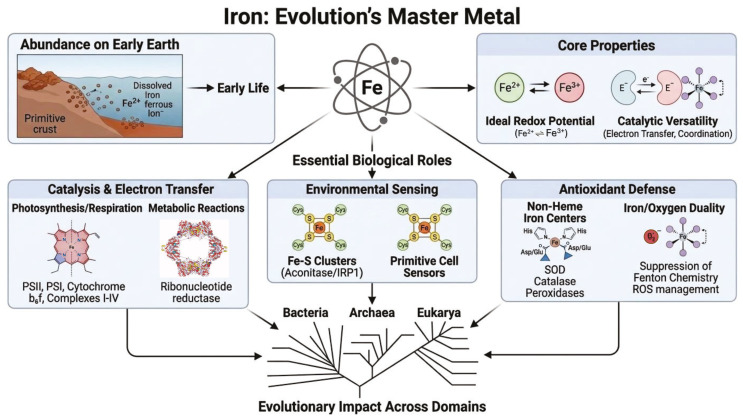
Iron as evolution’s master metal across all domains of life. Illustration showing iron’s unique properties and central biological roles, from catalysis and electron transfer to environmental sensing and antioxidant defense, underpins life’s reliance on this metal. Yet, iron reactivity requires tight cellular control, positioning it as both a vital engine and a potential hazard in modern biology. This figure was generated with the assistance of the AI-based tool FigureLabs.

Non-heme iron centers coordinate iron directly through amino acid side chains, typically histidine, aspartate, glutamate, or cysteine, without the scaffold of a porphyrin ring or the bridging sulfides of an Fe-S cluster [36]. This gives these centers the greatest geometric flexibility, where iron can adopt a wide range of coordination geometries. The absence of a rigid scaffold means that the protein environment has more influence over the metal’s reactivity. Mononuclear non-heme iron centers are found in a large family of dioxygenases and hydroxylases that use O_2_ as a substrate; dinuclear centers, in which two iron atoms are bridged by carboxylate and oxo groups, appear in enzymes as structurally different as ribonucleotide reductase, methane monooxygenase, and ferritin, all of which require two-electron redox chemistry that a single iron center cannot easily provide (Figure 3). Notably, heme biosynthesis itself in animals depends on iron–sulfur cluster chemistry: ferrochelatase, the terminal enzyme that inserts Fe^2+^ into protoporphyrin IX to complete heme synthesis in many organisms including humans, contains a [2Fe-2S] cluster essential for its catalytic activity [37]. This dependency of heme biosynthesis on a pre-existing Fe-S cluster enzyme further supports the hypothesis that iron–sulfur clusters represent the most ancient of the three cofactor classes.

#### 4.2.2. Iron in Photosynthesis

Photosynthesis depends on iron to an extent that is sometimes underappreciated. The photosynthetic apparatus of plants and cyanobacteria is among the most iron-dense systems in biology. For instance, chloroplasts in higher plants and photosynthetic membranes in cyanobacteria maintain millimolar iron concentrations, reflecting the metal’s pervasive involvement in light harvesting, electron transfer, and carbon fixation [38,39]. Iron limitation is one of the primary constraints on photosynthetic productivity in both marine and terrestrial environments, a point with direct relevance to the global carbon cycle.

The light-dependent reactions of oxygenic photosynthesis center on two large protein complexes, Photosystem I (PSI) and Photosystem II (PSII), connected by the cytochrome b_6_f complex. Although the water-oxidizing reaction at the heart of PSII (2H_2_O → O_2_ + 4H^+^ + 4e^−^) uses a manganese cluster as its catalytic center, iron is essential elsewhere in the same complex [40,41]. A non-heme iron atom positioned between the two plastoquinone binding sites (the Q_A_-Fe-Q_B_ center) facilitates electron transfer out of PSII, and cytochrome b559, a heme protein associated with PSII, plays a photoprotective role by providing an alternative electron transfer pathway that prevents the accumulation of oxidizing radicals when the normal photochemical cycle is disrupted (Figure 3).

The cytochrome b_6_f complex, which transfers electrons from PSII to PSI while coupling this transfer to proton translocation across the thylakoid membrane, contains four iron cofactors: two b-type hemes, one c-type heme, and a Rieske [2Fe-2S] cluster [42]. The Rieske cluster is unusual in that one of its iron atoms is coordinated by histidine rather than cysteine, which raises its midpoint potential relative to a standard [2Fe-2S] cluster and allows it to oxidize plastoquinol efficiently. The concerted action of these four iron centers drives the Q-cycle, a mechanism that effectively doubles the number of protons translocated per electron transferred and is central to the efficiency of photosynthetic ATP synthesis.

Photosystem I is the most iron-rich of the three complexes, containing three [4Fe-4S] clusters that relay electrons from the primary chlorophyll electron acceptor to ferredoxin, a small soluble iron–sulfur protein that in turn reduces NADP^+^ via ferredoxin- NADP^+^ reductase [43,44]. Ferredoxin operates at a midpoint potential of approximately—420 mV, among the most negative in biology, which provides the reducing power needed for carbon fixation, nitrogen assimilation, and the reductive regulation of Calvin cycle enzymes through thioredoxin. The fact that PSI requires three [4Fe-4S] clusters for what is essentially a single long-range electron transfer step reflects the demanding energetics of generating such a strongly reducing species from visible light.

One further aspect of iron in photosynthesis worth noting is the evolutionary flexibility in iron utilization observed in some photosynthetic organisms. In cyanobacteria and algae, the electron carrier between the cytochrome b_6_f complex and PSI can be either cytochrome c_6_, an iron-containing protein, or plastocyanin, a copper protein, depending on the availability of the respective metals. Under copper limitation, cells switch to cytochrome c_6_; under iron limitation, plastocyanin is favored [45,46]. This substitution is possible because both proteins occupy the same functional niche: they are both small, soluble, one-electron carriers with similar reduction potentials, but it requires maintaining the genetic and biosynthetic capacity for both. The flexibility illustrates how evolution can hedge against metal limitation in nutrient-poor environments.

#### 4.2.3. Iron in Aerobic Respiration

The mitochondrial electron transport chain is, in terms of iron content, even more demanding than the photosynthetic apparatus [47,48,49]. Complex I (NADH:ubiquinone oxidoreductase) alone contains eight Fe-S clusters that relay electrons from NADH through a series of precisely calibrated redox steps to ubiquinone, with midpoint potentials stepping from approximately—320 mV at the NADH end to approximately—45 mV at the ubiquinone end. The precise tuning of each cluster’s potential, achieved through differences in cluster type, protein environment, and hydrogen bonding to the sulfide bridges, ensures that electrons flow in the correct direction and that the energy released is used to drive proton translocation rather than dissipated as heat.

Complex II (succinate dehydrogenase) links the citric acid cycle directly to the respiratory chain, using three Fe-S clusters and a b-type heme to transfer electrons from succinate to ubiquinone. Complex III (the cytochrome bc1 complex or ubiquinol:cytochrome c oxidoreductase) operates by a Q-cycle mechanism analogous to that of the cytochrome b_6_f complex in photosynthesis, an evolutionary connection that reflects the common ancestry of these two complexes. Complex III contains two b-type hemes, one c-type heme, and a Rieske [2Fe-2S] cluster, and it transfers electrons from ubiquinol to the small soluble protein cytochrome c while pumping protons across the inner mitochondrial membrane. Complex IV (cytochrome c oxidase) completes the chain by reducing O_2_ to water, using two heme groups and two copper centers. The heme a_3_-CuB binuclear center at the active site of complex IV is where O_2_ binds and is reduced by four successive electron transfers from cytochrome c, with water as the only product (Figure 3).

Beyond the respiratory chain, iron serves a critical oxygen-carrying function in animals through hemoglobin and myoglobin. Both proteins use a single heme iron to bind O_2_ reversibly, a function that depends on maintaining the iron in the ferrous state: Fe^3+^ (methemoglobin) does not bind O_2_, which is why cells maintain methemoglobin reductases. In hemoglobin, the binding of O_2_ to one subunit increases the O_2_ affinity of the remaining subunits in a cooperative behavior that arises from the structural coupling between the four heme-containing subunits, allowing hemoglobin to load nearly completely at the O_2_ tension of the lungs and deliver most of its bound O_2_ at the lower tensions of respiring tissues [50]. Myoglobin, a monomeric protein in muscle, binds O_2_ with higher affinity than hemoglobin and serves primarily as a local O_2_ storage protein that can sustain aerobic metabolism during transient periods of high demand (Figure 3).

#### 4.2.4. The Chemical Range of Heme: Oxidases, Sensors, and Signal Transducers

The functional range of heme proteins extends well beyond O_2_ transport and electron transfer. The same heme cofactor, in different protein environments, can activate O_2_ for demanding oxidative chemistry, decompose reactive oxygen species, sense gaseous signals, or even generate them, a versatility that makes heme one of the most functionally diverse cofactors in biology.

Catalase uses heme iron to decompose H_2_O_2_ at rates that approach the diffusion limit, forming a high-valent Fe(IV)=O porphyrin radical intermediate (Compound I) that oxidizes a second molecule of H_2_O_2_ to O_2_) [51,52]. This remarkable catalytic efficiency (some catalases can process several million H_2_O_2_ molecules per second) reflects the tight optimization of the active site geometry and the electronic properties of the heme. Peroxidases use a similar high-valent iron intermediate to oxidize a wide range of organic substrates, with biological functions ranging from lignin biosynthesis in plant cell walls to the generation of hypochlorous acid by myeloperoxidase in neutrophils, a potent antimicrobial agent.

Cytochrome P450 enzymes constitute one of the largest enzyme superfamilies known, with more than 20,000 members identified across all domains of life [53,54]. They catalyze the insertion of one oxygen atom from O_2_ into an organic substrate (e.g., a monooxygenase reaction) with the other oxygen atom being reduced to water. The mechanism involves a sequence of electron transfers, substrate binding, O_2_ binding, O-O bond cleavage, and ultimately an ‘oxygen rebound’ step in which a high-valent Fe(IV)=O species (also called Compound I) transfers an oxygen atom to the substrate. P450s hydroxylate aliphatic and aromatic C-H bonds, including bonds that are among the most chemically inert in organic chemistry, and they are responsible in vertebrates for the metabolism of most drugs and xenobiotics, the biosynthesis of steroid hormones and bile acids, and the activation of vitamin D.

Nitric oxide synthase (NOS) uses heme to catalyze the oxidation of arginine to citrulline and nitric oxide, with NADPH and O_2_ as co-substrates. NO functions as a gaseous signaling molecule with diverse physiological roles: in blood vessels, it relaxes smooth muscle and regulates blood pressure; in the nervous system, it acts as a retrograde neurotransmitter; in the immune system, it contributes to the killing of intracellular pathogens The receptor for NO is soluble guanylyl cyclase, itself a heme protein: NO binds to the ferrous heme with high affinity, displacing the proximal histidine ligand and triggering a conformational change that activates cGMP synthesis. This NO-sGC-cGMP signaling axis is the target of nitro-vasodilator drugs and phosphodiesterase inhibitors [55,56]. Heme oxygenase, which degrades heme by cleaving the porphyrin ring to release CO and biliverdin (subsequently reduced to bilirubin), generates another gaseous signal, CO, which has been recognized more recently as a cytoprotective molecule with roles in vascular tone and anti-inflammatory signaling.

#### 4.2.5. Iron–Sulfur Clusters: Electron Carriers and Regulatory Switches

Iron–sulfur clusters participate in a wider range of biological functions than is sometimes appreciated. Their most obvious role (i.e., rapid one-electron transfer in respiratory and photosynthetic chains) reflects the quantum mechanical properties of the delocalized electron density across the iron and sulfide atoms, which allows very fast electron self-exchange rates. But Fe-S clusters also appear in enzymes that catalyze radical chemistry, in dehydratases that use the cluster as a Lewis acid catalyst rather than a redox center, in regulatory proteins that sense iron and oxygen availability, and in DNA repair and replication enzymes.

Mammalian cells contain two distinct aconitases: mitochondrial aconitase, which functions in the citric acid cycle [57], and cytosolic aconitase (also known as iron regulatory protein 1, IRP1), which resides in the cytoplasm, regulates intracellular iron homeostasis, and provides one of the most thoroughly studied examples of Fe-S cluster-mediated regulation [58]. When its [4Fe-4S] cluster is assembled and intact, the enzyme catalyzes the isomerization of citrate to isocitrate in the citric acid cycle by using the unique fourth iron of the cluster as a Lewis acid to bind and activate the substrate. When iron is limiting, or the cell is under oxidative stress, the cluster disassembles (i.e., the protein loses its labile fourth iron) and the resulting apoprotein acquires high-affinity RNA-binding activity, becoming iron regulatory protein 1 (IRP1). IRP1 then binds stem-loop structures called iron-responsive elements (IREs) in the 5′ or 3′ untranslated regions of mRNAs encoding iron metabolism proteins, stabilizing some mRNAs and blocking translation of others [59,60]. In this way, the assembly state of a single Fe-S cluster in aconitase/IRP1 serves as a direct sensor of cellular iron status, coupling metabolic and regulatory functions in a single protein.

Radical SAM (S-adenosylmethionine) enzymes represent one of the most functionally diverse enzyme families known, with more than 100,000 members predicted in sequenced genomes. All share a [4Fe-4S] cluster that reductively cleaves SAM to generate a 5′-deoxyadenosyl radical, a highly reactive species that abstracts a hydrogen atom from the substrate to initiate catalysis [61]. The downstream chemistry is extraordinarily varied: radical SAM enzymes carry out sulfur insertion, ring formation, methylation, isomerization, dehydration, and other reactions that would be difficult or impossible by polar mechanisms. They are involved in the biosynthesis of enzyme cofactors (including, recursively, Fe-S clusters themselves), in the modification of tRNA and rRNA, in the biosynthesis of natural products, and in the repair of DNA. Their distribution across all three domains of life and their phylogenetic diversity suggest that radical SAM chemistry is ancient.

#### 4.2.6. Non-Heme Iron Enzymes

Non-heme iron enzymes form a large and chemically diverse class whose unifying feature is the coordination of iron through protein-derived ligands such as histidine, aspartate, glutamate, cysteine, or tyrosine, rather than through the rigid scaffold of a porphyrin or the bridging sulfides of an Fe-S cluster [36]. This coordinative flexibility is reflected in the range of reactions these enzymes catalyze: from the hydroxylation of aromatic and aliphatic substrates by mononuclear dioxygenases, to the generation of the tyrosyl radical required for DNA synthesis by dinuclear ribonucleotide reductase, to the oxidative storage of iron in the ferritin nanocage.

The alpha-ketoglutarate-dependent dioxygenases are perhaps the largest and most physiologically consequential family among non-heme iron enzymes. They couple the oxidative decarboxylation of alpha-ketoglutarate to the hydroxylation of a substrate, with O_2_ providing both oxygen atoms [62,63]. The prolyl hydroxylases that modify hypoxia-inducible factor alpha (HIF-α)—signaling its proteasomal degradation under normoxia—are alpha-KG dioxygenases, as are the TET enzymes that oxidize 5-methylcytosine in DNA as part of epigenetic demethylation, and the Jumonji domain histone demethylases [64,65]. The activity of all these enzymes is therefore sensitive to iron availability, as well as to O_2_ and alpha-ketoglutarate concentrations, which means that iron status can directly influence gene expression through epigenetic mechanisms. This may help explain why iron deficiency has such broad effects on development and physiology.

Ribonucleotide reductase (RNR), which catalyzes the reduction of ribonucleotides to deoxyribonucleotides, is the rate-limiting step in DNA synthesis and is a dinuclear iron enzyme in class I bacteria and eukaryotes. The two iron atoms in the R_2_ subunit are used to generate and stabilize a tyrosyl radical that is then shuttled to the active site in the R1 subunit, where the actual substrate reduction occurs [66]. The coupling between the two subunits across a distance of roughly 35 Å, through a well-defined radical transfer pathway involving specific amino acid side chains, is one of the most striking examples of long-range radical chemistry in biology. Inhibiting RNR is a clinically validated strategy for treating cancer and viral infections; the drug hydroxyurea, for example, quenches the tyrosyl radical in the R2 subunit.

Methane monooxygenase (MMO), found in methanotrophic bacteria, uses a dinuclear iron center to activate O_2_ for the hydroxylation of methane to methanol. This is among the most difficult oxidation reactions in chemistry: the C-H bond of methane has a dissociation energy of approximately 440 kJ/mol, and breaking it under physiological conditions requires a highly reactive iron-oxo intermediate [67]. The mechanism involves the formation of a high-valent [Fe(IV)=O…Fe(IV)=O] or related species (compound Q) that abstracts a hydrogen atom from methane. The significance of this reaction, with methane being the most abundant hydrocarbon in natural gas and the simplest substrate for C1 chemistry, has motivated extensive efforts to understand and eventually mimic MMO’s chemistry in synthetic catalysts, with limited success so far. The ability of a protein-bound dinuclear iron center to perform this chemistry under ambient conditions, while fully synthetic systems require elevated temperatures and pressures, reflects the superior control that protein scaffolds can exert over metal reactivity.

In eukaryotic cells, Fe–S clusters are assembled primarily within mitochondria, making mitochondrial iron homeostasis essential not only for energy metabolism but also for nuclear genome regulation. Perturbations in mitochondrial iron availability influence the activity of nuclear Fe–S-dependent proteins involved in DNA methylation, histone demethylation, and chromatin remodeling, thereby providing a mechanistic basis for mitochondrial retrograde signaling through epigenetic regulation. Moreover, mitochondrial iron deficiency compromises the activity of RTEL1, an Fe–S-dependent DNA helicase required for telomere maintenance, resulting in telomere shortening and increased chromosomal instability [68]. These findings further emphasize that iron not only fuels cellular metabolism but also contributes to the maintenance of epigenetic identity and genome stability.

#### 4.2.7. Iron, Reactive Oxygen Species, and Antioxidant Defense

The same reactivity that makes iron such a useful catalyst is also the source of its most serious biological hazard. In the presence of H_2_O_2_ and O_2_, Fe^2+^ participates in Fenton chemistry (Fe^2+^ + H_2_O_2_ → Fe^3+^ + OH^−^ + OH^•^), generating hydroxyl radicals that react with DNA, proteins, and membrane lipids at near-diffusion-limited rates. The combination of the Fenton reaction with the superoxide-driven reduction of Fe^3+^ back to Fe^2+^ (the Haber–Weiss cycle) allows catalytic quantities of iron to generate sustained oxidative damage [31,69]. This is why cells maintain free iron concentrations at extraordinarily low levels and why virtually all iron in the cell is protein-bound at any given moment (Figure 3).

The iron-containing superoxide dismutases (Fe-SODs) address one part of this problem by disproportionating superoxide (2O_2_^•−^ + 2H^+^ → H_2_O_2_ + O_2_) at rates that approach the diffusion limit, thereby removing superoxide before it can reduce Fe^3+^ to Fe^2+^. Catalases and peroxidases then handle the H_2_O_2_ that SOD generates. Fe-SOD is found in prokaryotes and in the chloroplasts and mitochondria of eukaryotes, reflecting its probable origin in these organelles’ bacterial ancestors [70]. It is structurally similar to Mn-SOD, and the two can sometimes substitute for each other depending on metal availability, an example of the metalloenzyme plasticity noted in other contexts in this review.

The relationship between iron and oxygen in biology is thus best understood as one of managed tension. Iron is indispensable for the oxygen-dependent reactions of aerobic metabolism, yet oxygen amplifies iron’s toxicity by enabling Fenton chemistry. Cells have resolved this tension not by eliminating either reactant but by maintaining strict spatial and temporal control over both: iron is sequestered in storage proteins (ferritin) or bound in cofactors, and labile iron (the pool in transit between bound forms) is kept at concentrations too low to support significant Fenton chemistry under normal conditions [71]. When this control fails, as in iron overload disorders, the resulting oxidative damage is severe and often targets the liver, heart, and endocrine glands, organs with high iron content and metabolic activity.

## 5. Iron Homeostasis Across the Kingdoms of Life

### 5.1. The Central Problem

Every living cell faces a version of the same problem with iron: it needs enough to support metabolism but must prevent accumulation beyond what it can safely handle. The difficulty is that the threshold between sufficiency and toxicity is narrow, that the chemical environment determining iron availability can change rapidly, and that the mechanisms for acquiring, storing, and exporting iron all require energy and biosynthetic investment. The diversity of iron homeostasis systems across the tree of life, from the Fur repressor of bacteria to the hepcidin–ferroportin axis of vertebrates, reflects the variety of solutions that evolution has found to this shared constraint, each adapted to the particular ecological and metabolic context of the organism.

A common structural feature across these diverse systems is the use of iron-sensing proteins that couple iron availability to gene expression or post-translational regulation. In bacteria, proteins like Fur (Ferric Uptake Regulator) repress genes for iron acquisition when intracellular iron is elevated. While early models invoked direct Fe^2+^ coordination as the sensing mechanism [72], in vivo evidence for this is limited; more recent studies suggest that elevated intracellular iron promotes [2Fe-2S] cluster assembly in Fur, the cluster-loaded form of which functions as the active DNA-binding repressor, a mechanism analogous to that of IRP1 in eukaryotes [73]. The logic is simple: when iron is plentiful, the repressor is active, and uptake is suppressed; when iron is scarce, repression is relieved, and the uptake machinery is expressed. Eukaryotes use a functionally analogous but mechanistically distinct system in which iron regulatory proteins (IRPs) bind RNA structural elements (IREs) to control the translation and stability of mRNAs encoding ferritin, transferrin receptor, and other iron metabolism proteins, a post-transcriptional control layer that allows rapid responses to iron fluctuations without requiring new transcription [74]. Although the classical view describes Fur as a direct Fe^2+^-binding repressor, more recent in vivo evidence, however, suggests that the active DNA-binding form of Fur carries a [2Fe-2S] cluster assembled from elevated intracellular iron, a mechanism functionally analogous to IRP1 regulation in eukaryotes [72,73]. These models are not mutually exclusive since Fe^2+^ availability may govern cluster assembly, but the precise sensing mechanism remains an active area of investigation.

### 5.2. Microbial Iron Acquisition

In the aerobic, neutral-pH environments that most bacteria inhabit today, free Fe^3+^ is present at concentrations far too low, typically below 10^−17^ M at neutral pH, to support growth without active iron acquisition systems. The most widespread solution is siderophore secretion: small organic molecules with high affinity for Fe^3+^ are released into the environment, where they chelate iron from insoluble hydroxides, mineral surfaces, or host proteins, and the Fe^3+^-siderophore complexes are then recognized and imported by specific outer membrane receptors and periplasmic binding proteins. Over 500 structurally distinct siderophores have been described, falling into structural classes defined by their iron-chelating functional groups: catecholates (such as enterobactin from *E. coli*), hydroxamates (such as desferrioxamine B from Streptomyces), and mixed types [75,76].

**Figure 4:**
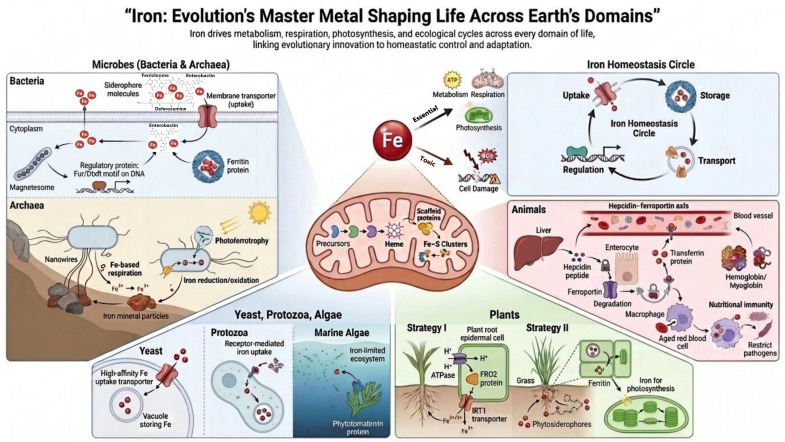
Iron’s central role in modern biology. Illustration depicting why life “chose” iron, highlighting its optimal redox potential, early Earth abundance, and catalytic versatility. Major iron cofactors—heme groups, iron–sulfur clusters, and non-heme iron centers—are shown driving key biological processes such as photosynthesis, respiration, and essential metabolic reactions. The figure includes Fe–S clusters as ancient catalysts and regulators (e.g., ISC/SUF systems and aconitase/IRP1), iron-dependent antioxidant defenses (SOD, catalase, peroxidases, and cytochrome P450), and the balance between iron’s vital roles in oxygen transport and energy production versus its potential for oxidative toxicity, controlled through cellular regulation and ferritin storage. This figure was generated with the assistance of the AI-based tool FigureLabs.

Enterobactin, the archetypal catecholate siderophore, binds Fe^3+^ with a stability constant of approximately 10^49^, which is among the highest measured for any chelator, and this exceptional iron affinity allows *E. coli* to scavenge iron from the host protein transferrin under conditions relevant to infection [75,76]. This same property has made enterobactin and its synthetic analogs of interest for designing iron-chelating therapeutics. Many bacteria produce multiple siderophores with different binding affinities and specificities, which may allow them to access different iron sources in complex environments. Some organisms bypass siderophore synthesis entirely by expressing receptors that recognize siderophores produced by other species, a strategy of chemical piracy that can provide a growth advantage in microbial communities where siderophore-producing species are abundant (Figure 4).

Under anaerobic conditions, where Fe^2+^ is the predominant form, many bacteria import iron directly through proton-coupled Fe^2+^ transporters, the most studied of which is the FeoABC system of *E. coli*. FeoB is a GTPase that couples GTP hydrolysis to Fe^2+^ transport across the cytoplasmic membrane, an unusual mechanism whose evolutionary logic is not fully clear but may reflect the need for a rapid, high-flux uptake system for the most bioavailable form of iron [77]. Several pathogens that cause intracellular infections, where iron may be available as Fe^2+^ in the reducing environment of phagosomes or the cytoplasm, rely heavily on Feo-type transporters.

### 5.3. Iron in Archaea and Extreme Environments

Archaea inhabiting hydrothermal vents, acid mine drainage, and other iron-rich extreme environments offer a window into the iron metabolisms that may have been widespread on the early Earth. Several archaeal and bacterial lineages use dissimilatory iron reduction, the coupling of organic matter oxidation to the reduction of Fe(III) minerals, as their primary energy-generating metabolism. This pathway, carried out by organisms such as *Geobacter* and *Shewanella* [78], involves the transfer of electrons from cytoplasmic NADH, through a series of c-type cytochromes and other carriers, to solid Fe(III) oxide phases outside the cell, a remarkable feat of long-range electron transfer that requires the bacterium to make physical contact with, or electrochemically connect to, an insoluble mineral. Dissimilatory iron reduction is among the most ancient metabolic strategies known, predating oxygenic photosynthesis, and it has profoundly influenced the iron geochemistry of anoxic sediments throughout Earth’s history (Figure 4).

The reverse process (i.e., chemolithotrophic iron oxidation, in which Fe^2+^ is oxidized to Fe^3+^ to provide the energy for CO_2_ fixation) is carried out by acidophiles such as *Acidithiobacillus ferrooxidans* at low pH, where Fe^2+^ remains stable in the presence of oxygen long enough to be used as an electron donor. At neutral pH, Fe^2+^ is oxidized abiotically so rapidly that only specialized microaerophilic bacteria can outcompete abiotic oxidation for the available Fe^2+^. Photoferrotrophy, the use of Fe^2+^ as the electron donor for anoxygenic photosynthesis, has been proposed as one of the mechanisms responsible for banded iron formation deposition in the Archean, because it would have oxidized Fe^2+^ to Fe^3+^ in a sunlit ocean before the evolution of oxygenic cyanobacteria [79,80].

Methanogens store iron in unusual thio-ferrate nanoparticles and rely on the SUF pathway for Fe-S cluster assembly, a pathway that is more resistant to oxidative inactivation than the ISC pathway used by many bacteria and eukaryotes, and that may represent the ancestral form of Fe-S cluster biosynthesis. Their iron regulation is mediated by DtxR-type metalloregulators that respond to intracellular Fe^2+^ rather than the Fur-type regulators of most bacteria, though the regulatory logic is similar [81]. The methane they produce, as a byproduct of CO_2_ reduction with H_2_, is itself a significant greenhouse gas, and the coupling of methanogenesis to iron reduction in anoxic sediments links iron cycling to the global carbon and methane budgets.

### 5.4. Regulation in Gram-Negative Bacteria: The E. coli Model

*E. coli* remains the best-characterized model for bacterial iron homeostasis, and its regulatory circuitry is worth describing in some detail because it illustrates principles that are conserved across many bacterial groups. The Ferric Uptake Regulator (Fur) is a global transcriptional repressor that, when intracellular iron is elevated, binds to ‘Fur box’ sequences in the promoters of genes for iron acquisition and represses their transcription [82]. When intracellular iron falls below a threshold, [2Fe-2S] cluster assembly in Fur is reduced, the repressor loses its DNA-binding activity, and iron acquisition genes are expressed. Fur regulates approximately 80 genes in *E. coli*, including those for siderophore biosynthesis and uptake, alternative iron-containing enzymes, and oxidative stress responses, a regulon that reflects the deep integration of iron metabolism with central physiology.

One of the more elegant regulatory mechanisms downstream of Fur involves the small RNA RyhB, which is expressed under iron-limited conditions when Fur is inactive [83]. RyhB base-pairs with and destabilizes mRNAs encoding non-essential iron-containing enzymes, including succinate dehydrogenase, fumarase, and several other TCA cycle enzymes, thereby releasing the iron bound in those proteins and reallocating it to more essential processes. This hierarchical regulation, in which a single transcription factor controls a small RNA that in turn controls a set of metabolic enzymes, allows the cell to prioritize iron use under scarcity without the delay associated with transcriptional reprogramming alone.

### 5.5. Evolutionary Adaptations to Iron Availability

Some bacteria have evolved remarkable adaptations that extend well beyond conventional iron uptake, storage, and utilization, illustrating the extraordinary evolutionary versatility of iron metabolism. Magnetotactic bacteria synthesize intracellular magnetic crystals, typically magnetite (Fe_3_O_4_) or greigite (Fe_3_S_4_), enclosed within lipid-bound organelles called magnetosomes [84]. These crystals are arranged in linear chains that function as a biological compass, allowing the bacteria to orient along the Earth’s magnetic field and navigate toward the optimal oxygen concentration in aquatic sediments. Magnetosome formation requires precise spatial and temporal control over iron uptake, intravesicular pH, and redox chemistry to produce single-domain crystals of the correct size and phase, and it involves a dedicated set of Mam proteins that organize the chain and control crystal growth [85]. The use of iron for a function unrelated to redox catalysis, namely navigation, illustrates how extensively evolution has exploited the metal’s unique physical properties.

At the other extreme, *Borrelia burgdorferi*, the causative agent of Lyme disease, has greatly minimized iron utilization with complete deletion of genes involved in iron metabolism [86]. It should be noted that *B. burgdorferi* is a highly specialized obligate parasite that evolved within an already iron-dependent biosphere and under the unusual selective pressure of a vertebrate host that actively sequesters iron as a form of nutritional immunity. Its reduced iron dependence therefore reflects a derived adaptation to a specific ecological niche, not a demonstration that complex life can originate or thrive independently of iron. Nonetheless, it has no detectable iron storage proteins, no iron–sulfur proteins, no iron-containing enzymes, and no known iron uptake systems or siderophore biosynthesis pathway. It has substituted manganese for iron in many enzymes that normally require iron. This evolutionary strategy likely reflects a combination of nutritional immunity, environmental metal availability, and evolutionary replacement by manganese-dependent pathways. By minimizing intracellular iron, these organisms may also reduce Fenton-mediated oxidative stress and the associated damage caused by iron-catalyzed reactive oxygen species.

Similar evolutionary reductions in iron utilization have been reported for *Treponema pallidum*, causative of syphilis, and several lactic acid bacteria, which have replaced many iron-dependent enzymes with manganese-dependent counterparts. Collectively, these examples demonstrate that iron requirements can be substantially reduced under highly specialized ecological conditions, although they do not challenge iron’s central role in the origin and evolution of life.

Beyond intracellular adaptations that either maximize or minimize iron utilization, microorganisms have also evolved sophisticated ecological strategies for competing over this essential metal within complex communities. Iron availability is a major determinant of microbial competition within complex communities, particularly in the mammalian gut microbiota. Members of the dominant bacterial taxa differ markedly in their iron requirements. Whereas members of the Lactobacillales and Bifidobacterium lineages have largely minimized their dependence on iron, many Proteobacteria and Enterobacteriaceae compete aggressively for environmental iron. Consequently, excess luminal iron favors the expansion of Proteobacteria at the expense of beneficial commensals [87]. Competition is further shaped by siderophore-mediated iron acquisition. In response, the host produces lipocalin-2, which sequesters enterobactin-type siderophores and selectively suppresses Enterobacteriaceae that depend on enterobactin, whereas pathogens producing lipocalin-2-resistant (“stealth”) siderophores retain a competitive advantage during intestinal inflammation [88].

## 6. Iron in Eukaryotes

### 6.1. Iron in Unicellular Eukaryotes

Unicellular eukaryotes (i.e., fungi, protozoa, and algae) introduced new structural and regulatory complexity to iron metabolism with the compartmentalization of cells into membrane-bound organelles. Mitochondria became the primary site of Fe-S cluster and heme biosynthesis, creating an obligate metabolic dependence on mitochondrial iron import that is conserved across all eukaryotes. This dependence links iron availability to mitochondrial function and, through that connection, to oxidative metabolism, cell growth, and apoptosis, relationships that are relevant to human pathology in contexts as different as mitochondrial iron overload in Friedreich’s ataxia and the iron dependence of rapidly dividing cancer cells.

In *Saccharomyces cerevisiae*, iron homeostasis has been studied in considerable detail. The high-affinity iron uptake system, which operates under iron limitation, uses a cell-surface ferrireductase (Fre1) to reduce Fe^3+^ to Fe^2+^, followed by a multicopper oxidase (Fet3) that immediately re-oxidizes the Fe^2+^ to Fe^3+^ as part of a Fe^3+^-specific permease (Ftr1) [89]. This oxidation-coupled transport mechanism, reducing iron to make it accessible, then immediately re-oxidizing it for transport, seems redundant at first glance but may reflect the specificity of the Ftr1 transporter for Fe^3+^ and the need to prevent the escape of free Fe^2+^ into the extracellular space where it could contribute to Fenton chemistry. Vacuoles serve as the primary iron storage compartment in yeast, substituting for the ferritin that most other eukaryotes use; iron is imported into the vacuole by Ccc1 and remobilized by a vacuolar permease under iron-limiting conditions.

In protozoan parasites, iron metabolism is intimately connected to virulence. *Trypanosoma brucei*, which causes African sleeping sickness, acquires iron by receptor-mediated endocytosis of host transferrin, using a surface receptor that is structurally unrelated to the mammalian transferrin receptor but serves the same function [90]. *Plasmodium falciparum*, the malaria parasite, obtains iron primarily by degrading host hemoglobin in the acidic food vacuole, and it detoxifies the released heme by polymerizing it into hemozoin, an inert crystalline form [91,92]. The dependence of these parasites on host iron has made iron acquisition pathways attractive drug targets, though translating this insight into clinically useful drugs has proved challenging.

Marine phytoplankton face chronic iron limitation in large areas of the open ocean, and they have evolved diverse and sometimes unexpected iron acquisition strategies in response. Some species use cell-surface ferrireductases and Fe^2+^ transporters; others take up colloidal iron or iron-organic complexes directly; diatoms express phyto-transferrin proteins that bind Fe^3+^ at the cell surface [93]. Under iron limitation, many algae remodel their photosynthetic apparatus to reduce iron demand, replacing the iron-rich cytochrome C6 with plastocyanin, degrading iron-rich light-harvesting complexes, and downregulating PSI, which contains by far the most iron of the three major photosynthetic complexes. The regulation of these acclimation responses and the diversity of iron acquisition systems across phytoplankton taxa remain active areas of research with implications for understanding marine primary productivity and the biological carbon pump.

### 6.2. Iron Acquisition in Plants

Plants face an iron acquisition challenge that differs from that of most other organisms: they are sessile, they obtain all their mineral nutrients from soil, and they must therefore work with whatever iron chemistry the soil presents. In well-drained, aerobic soils at neutral to alkaline pH, iron exists almost entirely as insoluble Fe(III) oxides and oxyhydroxides. In waterlogged, anoxic soils, Fe^2+^ can be present at phytotoxic concentrations. Plants must be capable of acquiring iron efficiently in the first type of soil while surviving the second, a range of conditions that has driven the evolution of flexible, multi-layered iron acquisition systems.

The strategy used by most dicots and non-grass monocots (Strategy I) involves three sequential steps at the root surface: acidification of the rhizosphere by plasma membrane H^+^-ATPases to solubilize Fe(III) minerals, reduction of solubilized Fe^3+^ to Fe^2+^ by the ferric reductase FRO2, and uptake of Fe^2+^ by the high-affinity iron transporter IRT1 [94]. All three components are transcriptionally induced under iron limitation, coordinated by the transcription factor FIT (Fe-deficiency Induced Transcription factor), which acts together with subgroup Ib bHLH proteins that integrate hormonal and metabolic signals [95,96]. Grasses use a different strategy (Strategy II) based on the synthesis and secretion of mugineic acid phytosiderophores from root cells, followed by uptake of the Fe^3+^-mugineic acid complex through YSL transporters. Strategy II is considerably more effective in calcareous soils and under low-temperature conditions, which may explain why grasses became the dominant vegetation in temperate grasslands.

Iron distribution within the plant involves two distinct chemical forms traveling through different vascular systems. In the xylem, iron moves upward as Fe^3+^ chelated by citrate; in the phloem, it moves as Fe^2+^ chelated by nicotianamine, a non-protein amino acid that is ubiquitous in plants and central to their metal homeostasis. The phloem pathway is particularly important for iron delivery to seeds, which must be provisioned with enough iron to support germination before the seedling can acquire iron from soil, and for the recycling of iron from senescing leaves back to the roots before leaf drop. In deciduous trees, the mobilization and resorption of leaf iron before autumn leaf abscission is a significant aspect of mineral nutrient cycling that is only beginning to receive attention.

Iron in plastids is required for chlorophyll synthesis (the enzyme that inserts magnesium into protoporphyrin IX requires iron for O_2_ activation in an earlier step), for the Fe-S clusters of the photosynthetic electron transport chain, and for ferredoxin. Excess iron in plastids is stored in ferritin, the same 24-subunit nanocage protein found in animals, though plant ferritins are targeted to plastids by a transit peptide and are regulated differently from their animal counterparts. Under iron deficiency, the most visible symptom is interveinal chlorosis, the yellowing of leaf tissue between veins while the veins remain green, reflecting the priority given to iron delivery through the vascular system and the relatively rapid collapse of chlorophyll levels when iron-dependent steps in chlorophyll biosynthesis fail.

### 6.3. Invertebrate Iron Metabolism

Invertebrates provide both a phylogenetic baseline and some striking specialized cases for understanding animal iron metabolism. The core machinery (e.g., ferrireductases converting Fe^3+^ to Fe^2+^, DMT1-type transporters importing Fe^2+^, ferritin storing Fe^3+^ [67], and the IRE-IRP system for post-transcriptional regulation) is broadly conserved across invertebrates, consistent with an origin early in animal evolution. What varies is how these components are deployed and what additional systems have evolved in response to particular life histories or dietary specializations.

*Drosophila melanogaster* has been particularly useful for understanding invertebrate iron metabolism in genetic terms. Iron enters through the anterior midgut via the Malvolio transporter (a DMT1 homolog) and is stored in ferritin or exported to the hemolymph. Unlike mammals, which use transferrin as the primary plasma iron carrier, *Drosophila* uses ferritin itself as a secreted iron carrier, whereby ferritin heavy chain (FHC) and light chain (FLC) are both secreted and circulate in the hemolymph loaded with iron. This use of ferritin as both a storage and transport protein may reflect the simpler circulatory system of insects, in which there is no equivalent of the vertebrate reticuloendothelial system for recycling iron from senescent red blood cells [97]. Iron dysregulation in *Drosophila*, achieved through mutations in iron metabolism genes or dietary manipulation, produces phenotypes including motor deficits, mitochondrial dysfunction, and neurodegeneration that have been used to model aspects of human diseases such as Parkinson’s and Friedreich’s ataxia.

Blood-feeding insects present a specialized iron management challenge: a single blood meal can deliver a dose of heme iron that would be toxic if not rapidly sequestered. Mosquitoes and ticks have evolved mechanisms to deal with this that include binding excess heme to specialized proteins, encapsulating it in peritrophic matrices that limit absorption, and inducing heme oxygenase to degrade it. In malaria parasites and related organisms, the equivalent strategy is polymerization of heme into hemozoin, an insoluble crystal and a detoxification strategy that has been exploited clinically, since the antimalarial drugs chloroquine and its analogs act in part by inhibiting hemozoin formation and allowing toxic heme to accumulate in the parasite.

### 6.4. The Hepcidin–Ferroportin Axis in Vertebrates

The most important advance in understanding vertebrate iron homeostasis over the past two decades has been the discovery and characterization of the hepcidin–ferroportin regulatory axis. Hepcidin is a small, cysteine-rich peptide hormone synthesized primarily by the liver; it binds to ferroportin, the only known cellular iron exporter, and triggers its internalization and degradation [98]. Because ferroportin is expressed on the basolateral surface of intestinal enterocytes (controlling iron absorption from the diet), on macrophages (controlling iron recycling from hemoglobin), and on hepatocytes (controlling iron release from stores), hepcidin can simultaneously regulate iron entry into the plasma from all three of these sources by adjusting ferroportin expression at each site (Figure 4).

The signals that regulate hepcidin expression reflect the major physiological contexts in which iron demand changes. When iron stores are high, hepatocytes sense elevated diferric transferrin saturation through cell surface complexes involving transferrin receptors 1 and 2 (TfR1, TfR2) and HFE and activate hepcidin expression through the BMP6/SMAD signaling pathway [99]. When erythropoietic demand for iron is high, such as after blood loss or at altitude, erythroid precursors secrete erythroferrone (ERFE), which suppresses SMAD signaling and reduces hepcidin, thereby allowing more iron to reach the bone marrow for hemoglobin synthesis. During infection and inflammation, IL-6 and other cytokines induce hepcidin through the JAK/STAT3 pathway, restricting iron availability to pathogens, a response termed ‘nutritional immunity’ that is also responsible for the anemia of chronic inflammation.

Mutations in the genes encoding hepcidin, ferroportin, or their upstream regulators cause hereditary iron overload disorders of varying severity [100]. Hereditary hemochromatosis type 1, the most common, results from mutations in HFE that impair transferrin sensing and lead to inappropriately low hepcidin relative to iron stores; iron accumulates over years in the liver, heart, and endocrine glands, causing cirrhosis, cardiomyopathy, and diabetes. The clinical management of hemochromatosis (i.e., therapeutic phlebotomy to remove excess iron) is effective if initiated before irreversible organ damage occurs, which argues for genetic screening in populations with high allele frequency for HFE mutations. Conversely, mutations that elevate hepcidin or reduce ferroportin function cause iron-refractory iron deficiency anemia (IRIDA), in which iron absorption is chronically suppressed even in the face of severe deficiency.

The evolutionary origin of the hepcidin–ferroportin system can be traced through comparative genomics. Ferroportin is present in all vertebrates and appears to be conserved in invertebrates as well, reflecting the fundamental importance of cellular iron export [93]. Hepcidin itself appears first in fish, suggesting that the endocrine coordination of systemic iron homeostasis is a vertebrate innovation, an adaptation perhaps associated with the evolution of a closed circulatory system and specialized red blood cells that demand large and tightly regulated iron fluxes for hemoglobin synthesis. Many teleost fish have two hepcidin genes, one primarily involved in iron regulation and one with antimicrobial activity, consistent with hepcidin’s origin as an antimicrobial peptide that was co-opted for iron regulation in early vertebrate evolution.

#### 6.4.1. Iron Across Vertebrate Taxa

The hepcidin–ferroportin system provides the systemic regulatory framework for iron homeostasis across vertebrates, but the specific demands and solutions vary considerably among different groups. In teleost fish, which represent the largest and most diverse vertebrate radiation, iron absorption occurs through both the intestine and the gills. Zebrafish have become a widely used genetic model for studying iron metabolism because of their optical transparency during embryogenesis, which allows the direct visualization of hemoglobin synthesis and iron distribution [101,102]. The zebrafish has provided insight into several aspects of iron metabolism, including the function of mitoferrin (the mitochondrial iron importer), the maturation of erythroid cells, and the zebrafish equivalents of human iron overload disorders.

Among the most striking vertebrate adaptations in iron metabolism are the Antarctic icefish, which have completely lost both hemoglobin and myoglobin through gene deletion [103,104]. These fish survive in the cold, well-oxygenated waters of the Southern Ocean by relying on the high physical solubility of O_2_ at low temperatures and on a suite of cardiovascular adaptations (e.g., enlarged heart, high blood volume, and elevated cardiac output) that compensate for the absence of hemoglobin. Their iron requirements are substantially lower than those of hemoglobin-expressing fish, yet the hepcidin–ferroportin regulatory system appears to be retained and functional, suggesting that iron management for non-hemoglobin purposes remains important even in these extreme cases.

Birds pose a different challenge: their high metabolic rates and sustained aerobic activity during flight demand efficient oxygen delivery and therefore high hemoglobin concentrations, placing heavy demands on iron metabolism. Iron is stored in eggs as vitelloferrin, and the chick’s developing hematopoietic system is supplied from this maternal store. Captive birds, fed diets richer in iron than their natural diets, frequently develop iron storage disease (i.e., progressive iron accumulation in parenchymal organs, particularly the liver), suggesting that the physiological mechanisms for excreting excess iron are insufficient when dietary supply is chronically elevated above what the species encountered during its evolutionary history.

In humans, the adult body contains approximately 3–4 g of iron, distributed primarily in hemoglobin (~70%), myoglobin (~10%), and storage forms including ferritin and hemosiderin (~20–30%). Daily absorption from the diet (1–2 mg) balances obligate losses through cell shedding and minor bleeding, and this balance is regulated primarily by hepcidin [105]. The prevalence of iron deficiency in human populations (estimated to affect over 2 billion people globally) is striking compared to other mammals and probably reflects a combination of factors specific to modern human lifestyles: high carbohydrate diets following the agricultural revolution that reduced heme iron intake, chronic blood loss from menstruation and parasitic infections, and the iron demands of frequent pregnancy. There is also reason to think that moderate iron deficiency may have provided some evolutionary protection against infections like malaria and tuberculosis, which depend on the host’s iron for their own metabolism.

#### 6.4.2. Coupling Iron and Oxygen Sensing

The connection between iron and oxygen sensing in vertebrate cells is mechanistically deep. The transcription factor HIF-1α, which orchestrates the cellular response to hypoxia, is regulated by a family of prolyl hydroxylase enzymes (PHDs) that mark HIF-1α for proteasomal degradation when O_2_ is available [62]. The PHDs are non-heme iron dioxygenases (e.g., their activity requires Fe^2+^ as a cofactor), meaning that iron deficiency mimics hypoxia by reducing PHD activity and allowing HIF-1α to accumulate. HIF-1α then activates a transcriptional program that includes, among other targets, erythropoietin (i.e., stimulation of red blood cell production), vascular endothelial growth factor (i.e., promotion of blood vessel growth), and transferrin receptor and DMT1, which increase cellular iron uptake. The integration of iron and oxygen sensing through a shared enzymatic mechanism ensures that cellular responses to these two limiting factors are coordinated rather than independent.

IRP2, the second of the two iron regulatory proteins, is degraded under iron-replete conditions by the FBXL5 ubiquitin ligase. FBXL5 contains an iron-binding hemerythrin-like domain that confers stability when iron and oxygen are both available but becomes unstable when either is limiting, again coupling iron and oxygen sensing in a single protein [106]. When FBXL5 is destabilized, IRP2 accumulates and activates the IRE-IRP post-transcriptional regulon, increasing TfR1 expression and repressing ferritin translation. This feedback architecture means that the cell’s iron acquisition machinery is coordinately regulated by both iron and oxygen availability, with responses calibrated to the demands of aerobic metabolism.

### 6.5. Ferroptosis: Iron-Dependent Cell Death and the Toll of an Iron-Based Chemistry of Life

The preceding sections argued that iron became indispensable to life precisely because of the chemical versatility of its Fe^2+^/Fe^3+^ couple: the same one-electron redox cycling that lets iron activate oxygen, shuttle electrons through the respiratory chain, and catalyze demanding enzymatic transformations also lets it react with peroxides to generate the hydroxyl radical. Ferroptosis is the cellular expression of that paradox. It is a regulated, non-apoptotic form of cell death driven by the iron-dependent accumulation of oxidized membrane phospholipids [107]. Unlike apoptosis, necroptosis, or pyroptosis, it is not executed by a dedicated effector machinery; rather, it emerges when the antioxidant systems that continuously restrain iron-catalyzed lipid peroxidation are overwhelmed [108,109]. Viewed through the theme of this chapter, ferroptosis is not an incidental pathology but the standing liability that aerobic, iron-using life has had to insure against: the entire glutathione/selenocysteine and NADPH-dependent defense apparatus described below is best read as the evolutionary toll levied for building a metabolism on Fenton-competent iron.

At the biochemical level, labile Fe^2+^ drives free-radical formation through Fenton chemistry and serves as the metal cofactor of the lipoxygenases that oxidize polyunsaturated fatty acids enzymatically. Once initiated, phospholipid hydroperoxides propagate self-amplifying peroxyl-radical chain reactions; the rate-limiting step is abstraction of a bis-allylic hydrogen from a polyunsaturated acyl chain, which is precisely why membrane lipid composition, rather than iron alone, dictates susceptibility. ACSL4 and LPCAT3 esterify arachidonic and adrenic acids into membrane phospholipids and thereby sensitize cells, whereas enrichment in monounsaturated fatty acids confers resistance [110,111]. Ferroptosis-like processes have been documented across evolutionary distant organisms, from yeast and algae to nematodes and plants, underscoring the ancient and inescapable relationship between iron-dependent metabolism and oxidative membrane damage [112]. A striking recent demonstration that this chemistry is genuinely rate-limiting comes from work showing that ferroptotic death can propagate across a tissue as a self-regenerating “trigger wave” of reactive oxygen species, travelling millimeter distances at constant speed rather than dissipating by diffusion [113]. Ferroptosis is therefore governed jointly by iron availability, lipid chemistry, and the reductive capacity that opposes radical propagation.

#### 6.5.1. Antioxidant Defenses Against Iron-Driven Lipid Peroxidation

Ferroptosis susceptibility is governed not by a single suppressor but by a network of antioxidant systems whose common currency is reducing power drawn ultimately from NADPH [108,114]. The canonical axis is GPX4, a selenocysteine-containing glutathione peroxidase that reduces phospholipid hydroperoxides to inert alcohols using glutathione that must be continually regenerated through cystine import, glutathione synthesis, and glutathione reductase [115]. The reliance on selenocysteine rather than ordinary cysteine reflects the evolutionary cost of maintaining this defense: cells invest in a dedicated, energetically expensive biosynthetic pathway to install a catalytically superior residue, an expenditure that is only justified if the peroxidation threat is constant.

GPX4 is, however, only the most studied member of a set of parallel, glutathione-independent defenses. FSP1 (formerly AIFM2) reduces coenzyme Q10 to ubiquinol at the plasma membrane and also drives a non-canonical vitamin K cycle that generates a potent lipophilic radical-trapping hydroquinone [116,117,118]. GTP cyclohydrolase-1 produces tetrahydrobiopterin (BH4), which traps lipid radicals and remodels the phospholipid pool toward peroxidation-resistant species [119]. A distinct surveillance arm was identified in MBOAT1 and MBOAT2, acyltransferases that remodel membrane phospholipids and are controlled by the estrogen and androgen receptors, making ferroptosis sensitivity a sex-hormone-dependent trait [120]. Because all of these arms ultimately consume NADPH, the metabolic machinery that supplies it becomes decisive. MESH1, a cytosolic NADPH phosphatase whose activity depletes NADPH and commits cells to ferroptosis, illustrates how the entire balance can be tipped by a single regulatory node [121]. The unifying principle is a single chemical economy: a layered set of radical trapping antioxidants (ubiquinol, vitamin K hydroquinone, BH4, reduced glutathione), each maintained in its active reduced state by a reducing budget that iron-driven peroxidation continuously tries to overdraw. Ferroptosis is what occurs when that budget is exhausted, and the elaborateness of the apparatus is a direct measure of how dangerous iron-based membrane chemistry is.

If the defense network sets the threshold for ferroptosis, iron-handling pathways set the offensive pressure against it. NCOA4-mediated ferritinophagy is pivotal: by selectively delivering ferritin to the lysosome, NCOA4 releases stored iron into the labile pool that drives Fenton chemistry and lipid peroxidation [122,123]. This axis thus functions as a regulated valve between safe iron storage and pathological iron-driven death, and its dysregulation is increasingly implicated in both neurodegenerative injury and tumor vulnerability. Recent work has further revealed that ferroptosis is, at bottom, a problem of competing lipid oxidation kinetics: two independent studies showed that 7-dehydrocholesterol (7-DHC), a cholesterol biosynthetic intermediate, is an endogenous suppressor of ferroptosis because its conjugated diene system reacts with peroxyl radicals far faster than polyunsaturated phospholipids do, thereby acting as a sacrificial radical sink that shields membranes from autoxidation [124,125].

#### 6.5.2. Ferroptosis in Disease and Its Broader Significance

Neurons are unusually exposed to ferroptosis owing to high oxygen consumption, polyunsaturated-lipid-rich membranes, and substantial regional iron loading. In Alzheimer’s and Parkinson’s diseases, iron accumulation, glutathione depletion, elevated ACSL4, and diminished GPX4 activity collectively shift neurons toward a ferroptosis-prone state, with comparable signatures observed in Huntington’s disease, multiple sclerosis, amyotrophic lateral sclerosis, and ischemia–reperfusion injury [126]. The causal involvement of lipid oxidation kinetics is supported by work showing that deuterium-reinforced polyunsaturated fatty acids, in which the rate-limiting bis-allylic hydrogen abstraction step is slowed by a kinetic isotope effect, reduce lipid peroxidation and improve outcomes in oxidative neurodegeneration models [127]. Translational efforts in this space have, however, underscored the importance of the defense-reinforcement approach over indiscriminate iron removal: the NRF2 activator omaveloxolone became the first FDA-approved therapy for Friedreich’s ataxia in 2023 [128], whereas iron chelation trials in neurodegeneration have produced cautionary results, illustrating that iron is metabolically indispensable and cannot simply be stripped from the brain without cost.

The same machinery can be pushed in the opposite direction in oncology: many tumors maintain elevated iron import and suppressed export, leaving them dependent on their ferroptosis defenses, and pharmacological inducers that deplete glutathione (class-1, e.g., erastin) or inhibit GPX4 directly (class-2, e.g., RSL3) exploit this vulnerability [129,130,131]. Because the defense network is redundant, durable therapeutic strategies are likely to require co-targeting of parallel arms rather than attack on a single node. Taken together, these observations return to a single point: ferroptosis sits at the intersection of iron metabolism, membrane chemistry, and redox biology because it is the unavoidable downside of the very property “facile one-electron redox cycling” that made iron the catalytic foundation of life. The selenocysteine peroxidase, the NADPH-fed radical-trapping antioxidants, ferritin storage, and ferritinophagic release are best understood not as a miscellany of pathways but as the integrated insurance system that aerobic life evolved to keep using iron without being destroyed by it.

## 7. Iron as a Constraint and a Catalyst in Evolution

Looking across the diversity of iron metabolism described in the preceding sections, several themes emerge that have broader implications for understanding biological evolution and the chemical constraints that shape it.

The first is that iron’s dominance in biology is not primarily a consequence of chemical superiority over all other transition metals; it is partly a consequence of historical availability. On the early Earth, Fe^2+^ was the most abundant soluble transition metal in the ocean by a wide margin, and the chemistry of the first metabolic systems was shaped by what was available. Other metals (e.g., copper, molybdenum, and nickel) became more important as geological and atmospheric conditions changed, and as organisms evolved the more elaborate acquisition and trafficking systems needed to use them. Iron’s early embedding in core metabolic processes made it difficult to replace even after conditions changed, and the subsequent evolution of increasingly sophisticated iron regulation systems represents an investment in managing a resource that biology had already committed to, rather than a freely chosen optimal solution.

The second theme is that iron’s toxicity (i.e., its tendency to catalyze Fenton chemistry in the presence of O_2_ and H_2_O_2_) has been as important a driver of biological innovation as its utility. The evolution of ferritin, siderophores, transferrin, hepcidin, and the many other components of iron homeostasis systems represents an enormous ongoing metabolic investment in controlling a reactive element. In this sense, iron has shaped not only enzyme active sites but also cellular organization (the compartmentalization of Fe-S cluster synthesis in mitochondria and chloroplasts), intercellular communication (hepcidin as a hormonal regulator of systemic iron distribution), and host–pathogen interactions (nutritional immunity as a defense strategy). Few elements have had such far-reaching effects on the architecture of living systems.

The comparison with other biologically important transition metals is instructive. Copper is redox-active and essential for aerobic metabolism (e.g., it appears in cytochrome c oxidase, in multicopper oxidases like Fet3 and ceruloplasmin, and in the copper–zinc superoxide dismutase of eukaryotes) but it is more toxic than iron at equivalent concentrations, more tightly regulated, and its adoption as a biological cofactor appears to have been later in evolution, coinciding with the rise in atmospheric oxygen that made Cu^2+^ more soluble. Manganese is used in the oxygen-evolving complex of PSII, in some SODs, and as an iron substitute in several enzymes in organisms like *B. burgdorferi* that have minimized iron use, but its slower kinetics and narrower redox range make it a less versatile general-purpose catalyst. Zinc is redox-inert but an excellent Lewis acid and structural cofactor, appearing in thousands of zinc finger proteins, zinc metalloproteases, and structural zinc sites in virtually every cell (but it cannot participate directly in electron transfer reactions, limiting its catalytic range). Despite iron’s dominant catalytic role, many biological iron pathways depend on cooperation with other metals. For example, multicopper ferroxidases are necessary for mammalian cellular iron export, high-affinity iron uptake in yeasts, and also in plants. In mammals specifically, three members of this family are known (e.g., ceruloplasmin (CP), hephaestin (HEPH), and zyklopen (ZP)), each containing six biosynthetically incorporated copper atoms that act as intermediate electron acceptors, coupling the oxidation of Fe^2+^ to the four-electron reduction of dioxygen to water [132]. The mechanistic logic behind this copper dependence is straightforward: ferroportin, the only known iron exporter in humans, releases iron as Fe^2+^, but transferrin, the major iron carrier in blood, can only bind Fe^3+^ effectively, making the ferroxidase step obligatory for iron to enter circulation. The three enzymes divide this labor across tissues: CP is particularly important for iron release from the liver and central nervous system, HEPH is the major MCF in the small intestine and is critical for dietary iron absorption, and ZP is involved in iron efflux from placental trophoblasts during iron transfer from mother to fetus [132]. In addition, copper works together with heme iron in the catalytic cycle of oxygen reduction in mitochondria.

Table 1 summarizes these comparisons across the four most biologically important transition metals.

### Future Directions: Iron and the Search for Life

Iron’s central role in terrestrial biochemistry raises an important question with profound implications for astrobiology and synthetic biology: to what extent is iron a necessary feature of life rather than a contingent consequence of Earth’s geochemical history? On Earth, iron availability shaped the development of core metabolism, and the specific cofactor chemistry that evolved around iron has proven extraordinarily difficult to replace. This suggests that iron-dependent biochemistry was not a random historical choice but reflected genuine advantages (i.e., redox accessibility, catalytic range, and early availability) that would be expected to favor iron in any life-generating environment with similar chemistry. If so, then planets with iron-rich geochemistry and persistent Fe^2+^/Fe^3+^ redox disequilibria might be disproportionately likely to host metabolically active biospheres.

Conversely, the existence of organisms like *B. burgdorferi* that have largely dispensed with iron demonstrates that iron-independent life is at least possible, if metabolically costly. On a planet depleted in iron, or one in which iron was unavailable in soluble form throughout the period when life was emerging, alternative metals might have been recruited instead. Whether such alternatives could support the same range of catalytic chemistry as iron, and whether they could enable comparable complexity, is an open question. Comparative studies of metal-limited model organisms, combined with astrobiological surveys for iron-bearing minerals and iron redox signatures in planetary environments, may help define the boundaries of iron’s necessity for life.

In synthetic biology, the constraints that iron imposes are becoming increasingly apparent as researchers attempt to engineer minimal cells and artificial metabolic systems. The need to manage iron and to provide enough for Fe-S cluster and heme biosynthesis while preventing Fenton chemistry adds a layer of complexity to any minimal-cell design that cannot easily be circumvented. Understanding precisely what iron management infrastructure is irreducibly necessary, and what can be simplified, is an important problem for the field. It is also, viewed from a different angle, a way of asking what the minimal requirements for life are, not in terms of genes or molecular machines, but in terms of the inorganic chemistry that those machines depend on.

Beyond astrobiology and synthetic biology, an important emerging research frontier concerns the impact of global environmental change on iron homeostasis. Growing evidence suggests that climate change may increasingly influence iron biology through both nutritional and infectious disease pathways. Elevated atmospheric CO_2_ concentrations reduce the iron content of major C_3_ staple crops, including wheat, rice, barley, and legumes, thereby increasing the risk of dietary iron deficiency in vulnerable populations [133]. At the same time, climate-driven shifts in the geographic distribution of vector-borne diseases may expose new populations to infection-induced hepcidin responses that suppress intestinal iron absorption and contribute to anemia of inflammation [134]. Although these interactions remain incompletely understood, they highlight the need to integrate iron homeostasis into future studies of climate resilience, nutrition, and global health, extending the influence of iron biology from the origin of life to the challenges facing modern civilization.

## 8. Conclusions

We began this review with the observation that iron connects the physics of stars to the chemistry of cells, and we have tried to show that this connection is more than a rhetorical device. Iron’s nuclear stability determines its cosmic abundance and therefore its abundance in planetary systems. Its geochemical behavior in the early Earth (e.g., soluble as Fe^2+^ under the anoxic conditions of the Archean ocean, abundant at hydrothermal vents, reactive on mineral surfaces) made it available to, and eventually embedded in, the first metabolic systems. The subsequent rise of O_2_ transformed its chemistry, imposed new demands on biological iron management, and forced the evolution of the regulatory infrastructure that now occupies a large fraction of every cell’s biosynthetic and signaling capacity.

Several themes cut across this account that seem worth making explicit. The first is that biological systems are constrained by chemistry in ways that are more fundamental than is sometimes appreciated. Iron is not central to metabolism because evolution chose it from among equally viable alternatives; it is central because the alternatives were genuinely less suitable given the conditions under which life emerged. The redox potential range of the Fe^2+^/Fe^3+^ couple, the availability of Fe^2+^ in the Archean ocean, and the catalytic versatility of iron in both one- and two-electron chemistry (e.g., these are not contingent historical facts but consequences of physics and planetary history). Understanding them helps explain why metabolism looks the way it does, and it provides a framework for thinking about what life elsewhere in the universe might look like.

The second theme is that iron’s toxicity has shaped biology as much as its utility. The elaborate iron homeostasis systems found in every living cell (e.g., siderophores, ferritin, transferrin, IRP/IRE regulation, and hepcidin) are not just incidental complexity but represent a sustained evolutionary response to the danger that iron poses in an oxygenated environment. Iron overload disorders, whether hereditary or acquired, illustrate what happens when these systems fail, and the spectrum of pathologies associated with iron dysregulation, from anemia at one extreme to hemochromatosis, neurodegeneration, and cancer at the other, reflects how tightly iron availability is coupled to cellular and organismal function.

The third theme is continuity. The chemistry that iron performs in modern enzymes is recognizably related to the chemistry it performed on mineral surfaces at hydrothermal vents; the Fe-S clusters in ferredoxin are structurally similar to the iron-sulfide minerals from which they may ultimately derive; and the principles that biology established billions of years ago continue to inform our understanding of iron redox chemistry.

What remains poorly understood is how much of what we see in iron biochemistry is truly necessary and how much is contingent. We do not know, for example, whether the specific coordination chemistry of the nitrogenase FeMo-cofactor is the only solution to biological nitrogen fixation, or merely the one that happened to arise and persist in the ancestor of all nitrogen-fixing bacteria. We do not know whether the hepcidin–ferroportin axis is the optimal solution to systemic iron regulation or one of many that would have worked equally well. We do not know whether life elsewhere, in an iron-rich environment similar to the early Earth, would converge on similar iron cofactor chemistry or find different solutions. These are not just academic questions. The answers would tell us something fundamental about the chemical boundary conditions of life, about what is constrained by physics and what is free to vary, and they might help guide both the search for life beyond Earth and efforts to engineer new biological or biomimetic systems here.

In the end, iron’s journey is a story about constraints and creativity; that is, how the fixed properties of a single element, operating through the laws of physics and chemistry, have shaped the structure of planets and the architecture of cells. The fact that the same element does all of these things is not a coincidence. It is what the physics demands.

## Figures and Tables

**Table 1:** Comparison of biologically important transition metals.

Property	Iron (Fe)	Copper (Cu)	Manganese (Mn)	Zinc (Zn)
Dominant redox states	Fe^2+^/Fe^3+^	Cu^+^/Cu^2+^	Mn^2+^/Mn^3+^/Mn^4+^	Zn^2+^ (redox-inert)
Redox potential range	Broad, biologically accessible (~−0.5 to +0.4 V)	Higher potentials (~+0.2 to +0.8 V)	Moderate, slower kinetics	None
Early Earth availability	High (soluble Fe^2+^ in anoxic oceans)	Low (poorly soluble)	Moderate	Moderate
Catalytic versatility	Very high: electron transfer, radical chemistry, small-molecule activation	High but hazardous at physiological concentrations	Moderate	Structural and Lewis acid only
Toxicity risk	High (Fenton chemistry)	Very high (strong oxidant)	Moderate	Low
Evolutionary role	Foundational, irreplaceable in core metabolism	Specialized, adopted later with O_2_ rise	Supportive, substitutive for iron in some lineages	Structural, regulatory, non-catalytic

## Data Availability

No new data were created or analyzed in this study. Data sharing is not applicable.
